# Computational simulation indicates that moderately high-frequency ventilation can allow safe reduction of tidal volumes and airway pressures in ARDS patients

**DOI:** 10.1186/s40635-015-0068-8

**Published:** 2015-12-10

**Authors:** Wenfei Wang, Anup Das, Oanna Cole, Marc Chikhani, Jonathan G. Hardman, Declan G. Bates

**Affiliations:** School of Engineering, University of Warwick, Coventry, CV4 7AL UK; Anaesthesia & Critical Care Research Group, School of Medicine, University of Nottingham, Nottingham, NG7 2UH UK

**Keywords:** Acute respiratory distress syndrome, Positive-pressure ventilation, Ventilator-induced lung injury, Computational simulation

## Abstract

**Background:**

A recent prospective trial using porcine models of severe acute respiratory distress syndrome (ARDS) indicated that positive-pressure ventilation delivered by a conventional intensive care ventilator at a moderately high frequency allows safe reduction of tidal volume below 6 ml/kg, leading to more protective ventilation. We aimed to explore whether these results would be replicated when implementing similar ventilation strategies in a high-fidelity computational simulator, tuned to match data on the responses of a number of human ARDS patients to different ventilator inputs.

**Methods:**

We evaluated three different strategies for managing the trade-off between increasing respiratory rate and reducing tidal volume while attempting to maintain the partial pressure of carbon dioxide in arterial blood (PaCO_2_) constant on a computational simulator configured with ARDS patient datasets.

**Results:**

For a fixed sequence of stepwise increases in the respiratory rate, corresponding decreases in tidal volume to keep the alveolar minute ventilation and inspiratory flow constant were calculated according to standard formulae. When applied on the simulator, however, these sequences of ventilator settings failed to maintain PaCO_2_ adequately in the virtual patients considered. In contrast, an approach based on combining numerical optimisation methods with computational simulation allowed a sequence of tidal volume reductions to be computed for each virtual patient that maintained PaCO_2_ levels while significantly reducing peak airway pressures and dynamic alveolar strain in all patients.

**Conclusions:**

Our study supports the proposition that moderately high-frequency respiratory rates can allow more protective ventilation of ARDS patients and highlights the potential role of high-fidelity simulators in computing optimised and personalised ventilator settings for individual patients using this approach.

**Electronic supplementary material:**

The online version of this article (doi:10.1186/s40635-015-0068-8) contains supplementary material, which is available to authorized users.

## Background

Acute respiratory distress syndrome (ARDS) is a severe condition that affects around 1 in 10,000 people every year with the mortality rate of 40–50 % [1, 2]. Mechanical ventilation (MV), involving the use of mechanical force to offload respiratory muscles of their work, is a fundamental component of treatment in the intensive care unit (ICU) for patients with ARDS. However, a problematic issue associated with MV is that it exposes patients’ lungs to potentially destructive energy applied by the ventilator [3]. Consequently, MV can induce lung injury and can increase the risk of non-pulmonary organ injury/failure, which further adds to morbidity and mortality for ARDS patients [4].

A number of studies have shown that lowering the tidal volume (*V*_T_) can improve mortality rates in ARDS patients. Hickling et al. [5] reported a 60 % decrease in the expected mortality rate among patients with ARDS by lowering *V*_T_. In another trial, Amato et al. [6] investigated changing conventional *V*_T_ (12 ml/kg of predicted body weight, PBW) to low *V*_T_ and reported a 46 % reduction in mortality. This benefit was also confirmed in the ARDS Network study with mortality decreased by 22 % in the low tidal volume intervention group [7]. However, reducing *V*_T_ by itself also leads to worsened partial pressures of arterial oxygen (PaO_2_) and carbon dioxide (PaCO_2_) and arterial pH [8].

An alternative approach to achieve more protective ventilation is high-frequency oscillatory ventilation (HFOV) [9]. In this approach, patients’ lungs are not allowed to exhale fully (keeping them partially inflated, which maintains oxygenation), while CO_2_ is cleared by moving small volumes of gas in and out of the respiratory system at 3 to 15 Hz (180 to 900 b/min). This process has the potential to minimise the repeated opening and collapsing of lung units that can cause secondary lung damage during mechanical ventilation [10]. Although HFOV is now a widely used lung-protective strategy in the treatment of neonatal and paediatric acute lung injury [11], it cannot be implemented on conventional ventilators, and clinical studies have so far failed to show a significant effect on mortality in adult patients undergoing mechanical ventilation for ARDS [12, 13].

A number of previous studies have also investigated the potential of moderately high-frequency ventilation using standard ventilators [14, 15]. In this approach, respiratory rates (RRs) applied are beyond the limits of traditional mechanical ventilation but below those used in HFOV. A recent prospective study using porcine models (*N* = 8) in which ARDS was induced by pulmonary lavage and injurious ventilation [16] supported the potential of moderately high-frequency ventilation to allow safe reductions in *V*_T_ and airway pressures while maintaining stable PaCO_2_ levels.

In this study, we explore whether the application of a similar approach using a high-fidelity computational simulator tuned to a number of human ARDS patient datasets confirms or refutes the results of this previous animal study. We also investigate a number of different approaches for practically implementing moderately high-frequency ventilation, by considering alternative algorithms for maintaining PaCO_2_ and reducing alveolar strain.

## Methods

### Computational simulator

The computational simulator used in this study is a multi-compartmental computational model that uses an iterative, time-sliced, arithmetic technique to simulate integrated respiratory and cardiovascular pathophysiological scenarios [17–19]. The core models in the simulator have been designed to represent a dynamic in vivo cardiovascular-pulmonary state using a set of mass-conserving equations based on well-established physiological principles. The model simulates a lung comprising conducting airways and 100 alveolar compartments, with each compartment having a corresponding set of parameters accounting for stiffness, threshold opening pressures (TOPs) and extrinsic pressures as well as airway and peri-alveolar vascular resistances. The mathematical principles and equations on which the simulator is based have been detailed in previous studies [20–22], which have also validated the simulator’s ability to represent the pulmonary disease states of individual patients with chronic obstructive pulmonary disease and ARDS. A detailed description of the principles and mathematical equations underlying the computational model implemented in our simulator is provided in Additional file 1.

### Model matching to ARDS patient data

The model was configured to match data from individual ARDS patients reported by Nirmalan and colleagues [23], which listed arterial and mixed venous blood gas values and cardiac output measurements taken from patients treated for ARDS. The problem of matching the model’s outputs to the patient data reported in [23] was formulated as an optimisation problem, where the difference between the simulated model outputs and the data is measured by a cost function. Internal model parameters are then varied by a numerical optimisation algorithm [24] in order to minimise this cost function. The cost function that captures the matching error between the model and the data is defined as:

1 $$ {E}_{\mathrm{T}}={\displaystyle \sum_{i=1}^n{E_i}^2} $$

where *E*_T_ is the total residual error representing the matching accuracy and ${E}_i=\frac{x_i-{x}_{id}}{x_{id}}$ is the error for output *i*, *x*_*i*_ is the value of output *i* returned by the simulation and *x*_*id*_ is the value of the data for that output. The model parameters for each compartment *i* used for the matching include threshold opening pressure TOP_*i*_, stiffness coefficient *S*_*i*_ and extrinsic pressure *P*_ext,*i*_, meaning that a total of 300 model parameters are considered for 100 compartments in the simulator. During the matching process, these parameters are allowed to vary continuously between physiologically realistic upper and lower bounds as defined in Table 1.

**Table 1:** Nominal values and allowable ranges for the model parameters

Parameters	Nominal value	Variation ranges
TOP_*i*_ (cmH_2_O)	30	(5, 60)
Stiffness coefficient *S*_*i*_ (cmH_2_O/ml^2^)	0.05	(0.005, 0.5)
Extrinsic pressure *P*_ext,*i*_ (cmH_2_O)	28.8	(−20, 28.8)

Global optimisation algorithms can then be used to find model parameter values that minimise the value of *E*_T_, i.e. minimise the difference between the model outputs and the data. The procedure is illustrated in detail in Additional file 1—in each iteration, a set of parameter combinations are sent to the simulator and the outputs from the simulator are evaluated by the optimisation algorithm which then generates the updated parameter values for the next iteration until the condition to get a best matching is found. In this study, we employed an advanced global optimisation algorithm known as a g*enetic algorithm*, a general-purpose, stochastic search and optimisation procedure, based on genetic and evolutionary principles [24]. Full details of the particular optimisation algorithm used in this study and how it was implemented with the model are also provided in Additional file 1.

### Strategies for implementing moderately high-frequency ventilation

After matching the model to the patient datasets, three different ventilation strategies were applied and evaluated separately on each of the virtual patients. The primary objective of the ventilation strategies was always to maintain a constant PaCO_2_ while increasing RR and reducing *V*_T_.

#### Strategy 1

The first two strategies attempt to calculate appropriate ventilator settings based on simple physiological equations that are widely used in clinical practice. To maintain PaCO_2_, patients are expected to have constant alveolar minute ventilation, which can be calculated using the following equation [25]:

2 $$ {M}_{\mathrm{V}}\mathrm{a}\mathrm{l}\mathrm{v}=\mathrm{R}\mathrm{R}\times \left({V}_{\mathrm{T}}-{V}_{\mathrm{D}}\mathrm{a}\mathrm{nat}\right) $$

In Eq. (2), *M*_V_alv is the alveolar minute ventilation, RR is the respiratory rate, *V*_T_ is the tidal volume and *V*_D_anat is the anatomical dead space. To investigate the effect of higher frequency ventilation, the ventilator rate RR for each of the virtual patients is increased from 16 to 48 b/min in steps of 8 b/min, with each step lasting for 20 min. At each step, the corresponding *V*_T_ is reduced according to Eq. (2) above, so as to maintain constant alveolar minute ventilation. *V*_D_anat can be estimated based on the ideal body weight [26]; in this study, we used a value of 160 ml, based on an adult patient with ideal body weight of 70 kg.

#### Strategy 2

Here, as well as keeping alveolar minute ventilation constant using Eq. (2), the inspiratory flow is also kept constant (this corresponds to the strategy implemented in [16]). This can be achieved in our simulator by using the equation [25]:

3 $$ {F}_{\mathrm{insp}}=\frac{V_{\mathrm{T}}\times \mathrm{R}\mathrm{R}}{60\mathrm{D}\mathrm{C}} $$

where *F*_insp_ is the inspiratory flow into the lung from the ventilator and DC is the duty cycle (inspiratory time divided by total cycle time). From Eq. (3), by varying DC, *F*_insp_ can be manipulated since *V*_T_ is already determined by Eq. (2). Thus, the difference between strategies 1 and 2 is that DC is set as constant for the first, while for the latter, DC is varied to achieve constant inspiratory flow.

#### Strategy 3

An alternative approach to computing changes in ventilator settings based on simple physiological equations is to exploit the computational simulator directly. In this approach, we use numerical optimisation to calculate the value of *V*_T_ at each increment of RR that will minimise the change in the value of PaCO_2_. For each value of RR from 16 to 48 b/min at each step, the corresponding values of *V*_T_ (denoted by *p*_1_, *p*_2_,…, *p*_5_) are selected by an optimisation algorithm between a lower bound of 2.5 ml/kg and an upper bound of 8 ml/kg. A cost function is defined as the difference between the model-generated values and the initial value of PaCO_2_. During the optimisation process, the values of *p*_*i*_ are considered as optimisation variables that are systematically varied within the bounded space until the values that minimise the cost function are found. The process is then repeated for all three patient models.

## Results

### Matching the simulator to ARDS patient datasets

To configure the virtual ARDS patients, three sets of patient data from Nirmalan’s study [23] were used, which are classified as mild (patient A, partial pressure of arterial oxygen/fraction of inspired oxygen (PaO_2_/FIO_2_) ratio 253.9 mmHg), moderate (patient B, PaO_2_/FIO_2_ ratio 166.3 mmHg) and severe (patient C, PaO_2_/FIO_2_ ratio 55.6 mmHg) according to the ARDS Berlin definition [27]. The initial *V*_T_ was set at 8 ml/kg (assuming ideal body weight of 70 kg), and RR was set to 12.5 b/min for all three patients. The cardiac output (CO), FiO_2_ and haemoglobin (Hb) were configured using the data reported in the reference. Pulmonary vascular resistance (PVR) and compliance used for the model are also reported in Table 2. The model outputs to be matched included PaO_2_, partial pressure of oxygen in mixed venous blood (PvO_2_), partial pressure of carbon dioxide in the venous blood (PvCO_2_) and pulmonary shunt fraction. The initial value of PaCO_2_ was produced by the model at the matched state. An optimisation algorithm was run to minimise the difference between model outputs and the data based on Eq. (1), until a predefined termination criterion was reached. The model-fitting results are reported in Table 2; this shows that close matches to each of the three patient datasets were achieved by the simulator.

**Table 2:** Model fitting for three ARDS patients

	Patient A		Patient B		Patient C	
CO (l/min)	13.0		10.1		3.9	
FIO_2_	0.8		1		1	
Hb (g/l)	112		79		98	
PVR (dyn s cm^−5^)	152.0		152.1		151.9	
*C*_dyn_ (ml/mbar)	37		31		33	
DC (ratio)	0.33		0.33		0.33	
RR (b/min)	12.5		12.5		12.5	
	Data	Model	Data	Model	Data	Model
PaO_2_ (mmHg)	201	203.1	165.75	166.29	56.32	55.59
PvO_2_ (mmHg)	45.83	47.68	42	41.51	24.83	25.66
PvCO_2_ (mmHg)	46.73	43.34	43.5	43.61	39.3	39.79
Shunt (% of CO)	30.0	30.8	33.5	34.7	34.7	35.21

### Effectiveness of the three ventilation strategies

**Fig. 1:**
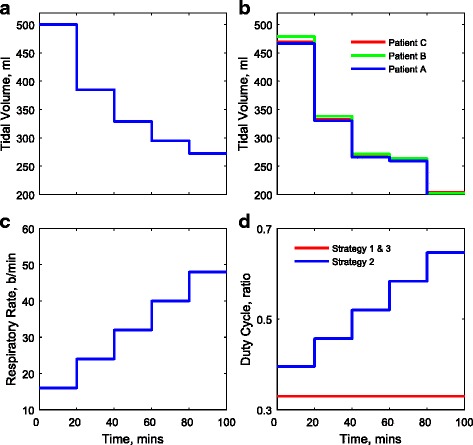
Mechanical ventilator settings for each ventilation strategy. **a** *V*_T_ for strategies 1 and 2. **b** *V*_T_ for strategy 3. **c** RR for all strategies. **d** DC for each strategy

**Fig. 2:**
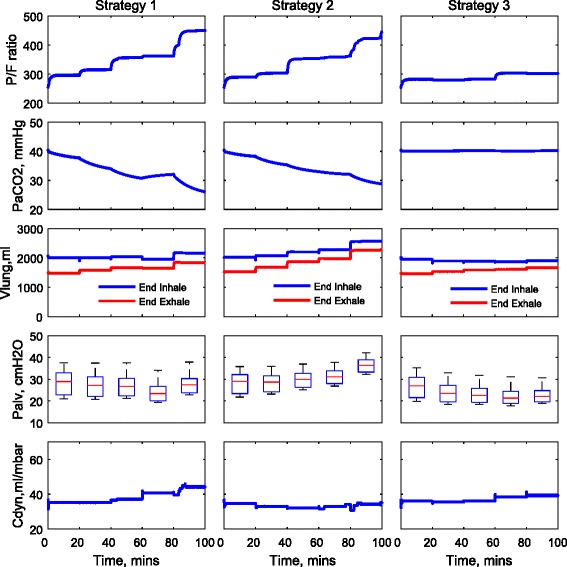
Model outputs for patient A under different ventilation strategies

**Fig. 3:**
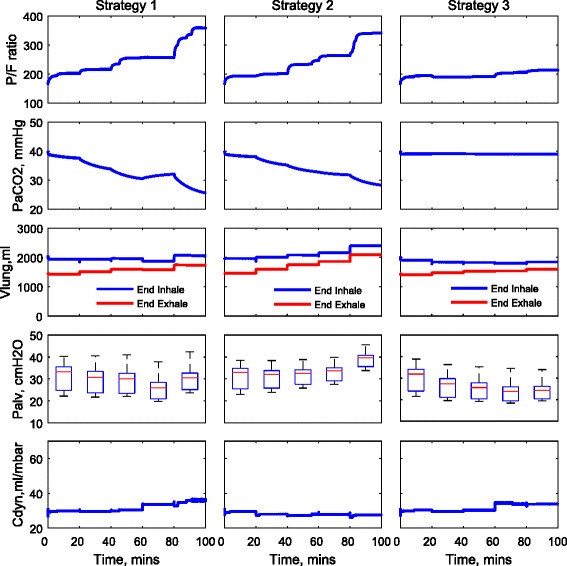
Model outputs for patient B under different ventilation strategies

**Fig. 4:**
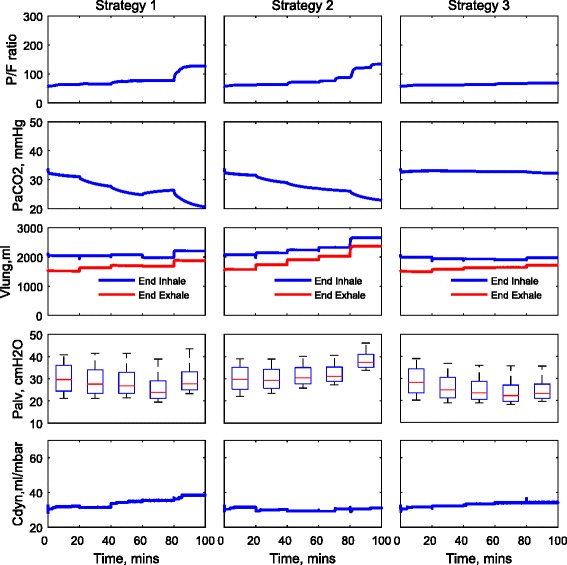
Model outputs for patient C under different ventilation strategies

**Fig. 5:**
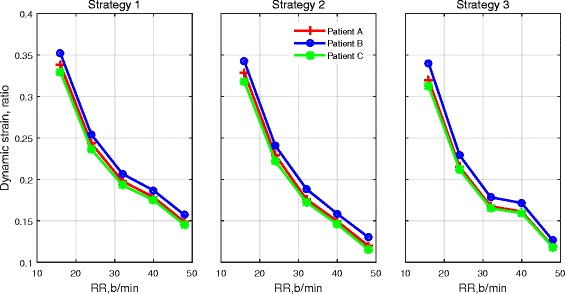
Dynamic alveolar strain (*V*_T_/FRC) for each patient as RR increased under the different ventilation strategies

The ventilation settings used for each strategy are reported in Table 3 and are also shown in Fig. 1. For strategies 1 and 2, the same settings for *V*_T_ are applied to each patient; see Fig. 1a. For strategy 3, the optimal values of *V*_T_ are computed for each patient using numerical optimisation; see Fig. 1b. Figure 1c shows the same pattern of RR applied for all strategies, and the variations of DC are shown in Fig. 1d. The corresponding changes of the P/F ratio, PaCO_2_, peak alveolar pressure (*P*_alv_, calculated from the 25 % of alveoli with highest pressures), dynamic compliance (*C*_dyn_) and the volume of the lung at the end of inhalation and exhalation (*V*_lung_) for each patient are shown in Figs. 2, 3 and 4. For all patients, strategies 1 and 2 led to significant changes in PaCO_2_, whereas strategy 3 kept the value of PaCO_2_ almost constant for all combinations of *V*_T_ and RR. The changes in PaCO_2_ observed for strategies 1 and 2 are accompanied by large increases in the P/F ratio (due to alveolar recruitment arising from increases in *P*_alv_)—only strategy 3 showed a consistent reduction in *P*_alv_ as RR increased, i.e. only strategy 3 implemented a more *protective* ventilation strategy. The potential of moderately high-frequency ventilation to deliver more protective ventilation is further supported by Fig. 5, where dynamic strain (*V*_T_/FRC, ratio) can be seen to decrease most significantly in all three simulated patients as RR is increased using strategy 3.

**Table 3:** Ventilation settings for strategies 1 and 2

RR (b/min)	16	24	32	40	48
*V*_T_ (ml)	497.5	385	328.75	295	272.5
*V*_D_anat (ml)	160	160	160	160	160
*M*_V_alv (ml/min)	5400	5400	5400	5400	5400
DC (ratio) (strategy 1)	0.33	0.33	0.33	0.33	0.33
DC (ratio) (strategy 2)	0.40	0.46	0.52	0.58	0.65
*F*_insp_ (ml/s) (strategy 1)	404.04	466.67	531.31	595.96	660.61
*F*_insp_ (ml/s) (strategy 2)	337	337	337	337	337

## Discussion

**Fig. 6:**
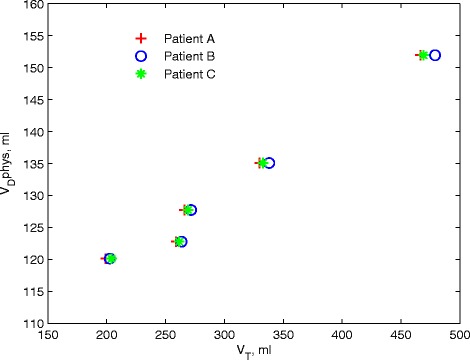
Relationship between *V*_D_phys and *V*_T_ for each patient using strategy 3

Why do strategies 1 and 2 result in large changes in PaCO_2_ when they are predicated on maintaining constant alveolar minute ventilation and inspiratory flow? The answer is provided by Fig. 6, which plots the relationship between the total dead space (physiological dead space, *V*_D_phys) calculated in the simulation model and *V*_T_ for each virtual patient. As shown, as RR increases, the value of *V*_D_phys is not constant, as assumed in Eqs. (2) and (3) using *V*_D_anat (on which strategies 1 and 2 are based), but is strongly proportional to *V*_T_. It may also be observed that the ratio *V*_D_phys/*V*_T_ is similar through the spectrum of ARDS severity. Interestingly, this relationship has previously been shown experimentally in the context of high-frequency ventilation [28]. Thus, the simple formulae on which strategies 1 and 2 are based do not correctly compute the required changes in *V*_T_ and RR to keep alveolar minute ventilation constant with either constant or variable inspiratory flow.

**Fig. 7:**
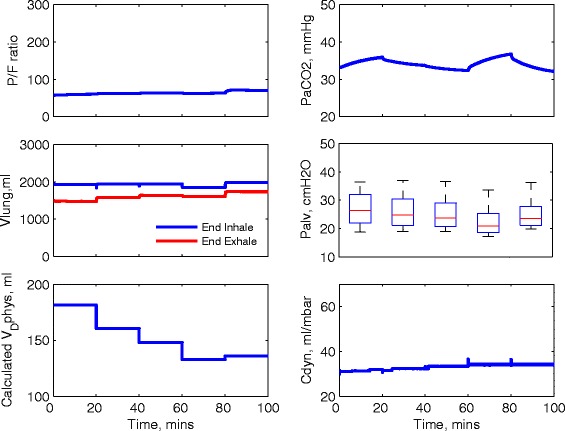
Model outputs for patient C using strategy 1 with calculated *V*_D_phys

A test was performed using the model that calculated *V*_D_phys instead of *V*_D_anat during each step to estimate which *V*_T_ should be applied in the next change of RR to maintain *M*_V_alv based on Eq.(2). The simulation results for patient C using strategy 1 with the calculated *V*_D_phys are shown in Fig. 7. It can be seen that although PaCO_2_ was not constant, it remained within an acceptably narrow range, which is significantly improved compared with the results in Fig. 4. In practice, therefore, if a device could provide a measurement of *V*_D_phys in a sufficiently short time, our results indicate that PaCO_2_ could be well controlled using this approach.

The benefits of a normal or only slightly elevated level of PaCO_2_ in critically ill patients are well recognised. It is, however, difficult and sometimes impossible to achieve this in a patient with ARDS without increasing the risk of alveolar injury. The concept of permissive hypercapnia is now an accepted management strategy for the critically ill lung. The rationale behind this approach is primarily to minimise lung strain, which could otherwise be worsened by strenuous ventilatory strategies aimed at keeping PaCO_2_ within normal physiological limits. However, in a very severe lung disease, the rise in PaCO_2_ is sometimes difficult to control using conventional protective ventilation strategies. Extracorporeal CO_2_ removal devices have been used in clinical practice for a few years now, but they bring potentially dangerous side effects [29]. The efficacy of these devices in allowing ultra-protective ventilation strategies, with tidal volumes and pressures comparable to those used in our model simulation, is being tested at present in the SUPERNOVA trial [30].

The benefit of the moderately high-frequency ventilation strategies we tested in this model is that they allow the maintenance of a normal level of PaCO_2_ while seeming to have the potential for decreasing the risk of lung injury and, in some cases, while recruiting the lung. The problem of improving lung protection in ARDS is obviously an extremely complex and multifaceted one and involves consideration of multiple factors beyond mechanical pressure limits. Nonetheless, our results indicate that moderately high-frequency ventilation could represent a beneficial ventilation strategy in ARDS patients with reduced respiratory system compliance. An interesting question for future investigation is to determine the potential for reducing driving pressure using this strategy, since a recent retrospective analysis of nine randomised trials indicated that driving pressure was the variable most strongly associated with survival [31]. Our study has a number of limitations, principally the small number of “virtual” patients evaluated and the lack of prospective validation in clinical trials. We note, however, that our findings confirm and help to explain the results of a previous study using animal models that investigated very similar changes in ventilator settings [16].

## Conclusions

By using a high-fidelity computational simulator, a more protective ventilation strategy consisting of progressive reductions in *V*_T_ with simultaneous increases in RR could be developed for a number of different virtual ARDS patients, covering the spectrum from mild to severe presentations of the disease. This strategy allowed changes in PaCO_2_ to be kept within an acceptable range in each case, thus confirming the results reported on a cohort of porcine models in [16], which showed that moderately high-frequency ventilation could allow safe reductions in the levels of *V*_T_. Attempts to compute appropriate values for the decrements in *V*_T_ required to compensate for increments in RR using simple mathematical formulae were not successful, due to the fact that dead space varies significantly with changes in *V*_T_. Our results demonstrate the importance of using advanced simulation models in order to correctly represent the complex dynamics of ventilator-lung interactions and highlight the potential of such models to refine or replace animal trials in this area.

## Additional file

Additional file 1:
**Simulation model description and optimisation algorithm description.**

## References

[1] Villar J, Blanco J, Añón JM, Santos-Bouza A, Blanch L, Ambrós A, Gandía F, Carriedo D, Mosteiro F, Basaldúa S (2011). The ALIEN study: incidence and outcome of acute respiratory distress syndrome in the era of lung protective ventilation. Intensive Care Medicine.

[2] Erickson SE, Martin GS, Davis JL, Matthay MA, Eisner MD (2009). Recent trends in acute lung injury mortality: 1996–2005. Critical Care Medicine.

[3] Zilberberg MD, de Wit M, Pirone JR, Shorr AF (2008). Growth in adult prolonged acute mechanical ventilation: implications for healthcare delivery. Critical Care Medicine.

[4] Slutsky AS, Tremblay LN (1998). Multiple system organ failure: is mechanical ventilation a contributing factor?. American Journal of Respiratory and Critical Care Medicine.

[5] Hickling K, Henderson S, Jackson R (1990). Low mortality associated with low volume pressure limited ventilation with permissive hypercapnia in severe adult respiratory distress syndrome. Intensive Care Medicine.

[6] Amato MBP, Barbas CSV, Medeiros DM, Magaldi RB, Schettino GP, Lorenzi-Filho G, Kairalla RA, Deheinzelin D, Munoz C, Oliveira R (1998). Effect of a protective-ventilation strategy on mortality in the acute respiratory distress syndrome. New England Journal of Medicine.

[7] Network ARDS (2000). Ventilation with lower tidal volumes as compared with traditional tidal volumes for acute lung injury and the acute respiratory distress syndrome. New England Journal of Medicine.

[8] Fuchs H, Mendler MR, Scharnbeck D, Ebsen M, Hummler HD (2011). Very low tidal volume ventilation with associated hypercapnia-effects on lung injury in a model for acute respiratory distress syndrome. PloS One.

[9] Derdak S, Mehta S, Stewart TE, Smith T, Rogers M, Buchman TG, Carlin B, Lowson S, Granton J, Investigators MOVFARDSTS (2002). High-frequency oscillatory ventilation for acute respiratory distress syndrome in adults: a randomized, controlled trial. American Journal of Respiratory and Critical Care Medicine.

[10] Riphagen S, Bohn D (1999). High frequency oscillatory ventilation. Intensive Care Medicine.

[11] Graciano AL, Freid EB (2002). High-frequency oscillatory ventilation in infants and children. Current Opinion in Anesthesiology.

[12] Young D, Lamb SE, Shah S, MacKenzie I, Tunnicliffe W, Lall R, Rowan K, Cuthbertson BH (2013). High-frequency oscillation for acute respiratory distress syndrome. New England Journal of Medicine.

[13] Ferguson ND, Cook DJ, Guyatt GH, Mehta S, Hand L, Austin P, Zhou Q, Matte A, Walter SD, Lamontagne F (2013). High-frequency oscillation in early acute respiratory distress syndrome. New England Journal of Medicine.

[14] Eriksson I, Sjöstrand U (1980). Effects of high-frequency positive-pressure ventilation (HFPPV) and general anesthesia on intrapulmonary gas distribution in patients undergoing diagnostic bronchoscopy. Anesthesia & Analgesia.

[15] Barzilay E, Lev A, Ibrahim M, Lesmes C (1987). Traumatic respiratory insufficiency: comparison of conventional mechanical ventilation to high-frequency positive pressure with low-rate ventilation. Critical Care Medicine.

[16] Cordioli RL, Park M, Costa ELV, Gomes S, Brochard L, Amato MBP, Azevedo LCP (2014). Moderately high frequency ventilation with a conventional ventilator allows reduction of tidal volume without increasing mean airway pressure. Intensive Care Medicine Experimental.

[17] Hardman J, Wills J (2006). The development of hypoxaemia during apnoea in children: a computational modelling investigation. British Journal of Anaesthesia.

[18] McCahon R, Columb M, Mahajan R, Hardman J (2008). Validation and application of a high-fidelity, computational model of acute respiratory distress syndrome to the examination of the indices of oxygenation at constant lung-state. British Journal of Anaesthesia.

[19] Hardman J, Bedforth N, Ahmed A, Mahajan R, Aitkenhead A (1998). A physiology simulator: validation of its respiratory components and its ability to predict the patient’s response to changes in mechanical ventilation. British Journal of Anaesthesia.

[20] Das A, Menon PP, Hardman JG, Bates DG (2013). Optimization of mechanical ventilator settings for pulmonary disease states. Biomedical Engineering, IEEE Transactions on.

[21] Wang W, Das A, Ali T, Cole O, Chikhani M, Haque M, Hardman JG, Bates DG (2014). Can computer simulators accurately represent the pathophysiology of individual COPD patients. Intensive Care Med Exp.

[22] Das A, Cole O, Chikhani M, Wang W, Ali T, Haque M, Bates DG, Hardman JG (2015) Evaluation of lung recruitment maneuvers in acute respiratory distress syndrome using computer simulation. Critical Care 19 (1):1–15 10.1186/s13054-014-0723-6 PMC4329196 25578295

[23] Nirmalan M, Willard T, Columb M, Nightingale P (2001). Effect of changes in arterial-mixed venous oxygen content difference (C (a–v̄) O2) on indices of pulmonary oxygen transfer in a model ARDS lung. British Journal of Anaesthesia.

[24] Golberg DE (1989) Genetic algorithms in search, optimization, and machine learning. Addison Wesley, Boston, MA, USA

[25] West JB (2012). Respiratory physiology: the essentials.

[26] Ganong WF, Barrett KE (2005). Review of medical physiology.

[27] Force ADT (2012). Acute respiratory distress syndrome. JAMA.

[28] Chakrabarti M, Gordon G, Whitwam J (1986). Relationship between tidal volume and deadspace during high frequency ventilation. British Journal of Anaesthesia.

[29] Brogan TV, Thiagarajan RR, Rycus PT, Bartlett RH, Bratton SL (2009). Extracorporeal membrane oxygenation in adults with severe respiratory failure: a multi-center database. Intensive Care Medicine.

[30] SUPERNOVA trial of ECCO2-R for moderate ARDS. http://www.esicm.org/research/trials-group/supernova. Accessed November 2015

[31] Amato MB, Meade MO, Slutsky AS, Brochard L, Costa EL, Schoenfeld DA, Stewart TE, Briel M, Talmor D, Mercat A (2015). Driving pressure and survival in the acute respiratory distress syndrome. New England Journal of Medicine.

